# Green Composite Based on a Polymer Mixture Containing Biopolymer and Waste Coffee Husks

**DOI:** 10.3390/polym17131748

**Published:** 2025-06-24

**Authors:** Cezary Gozdecki, Marek Kociszewski, Krzysztof Moraczewski, Tomasz Karasiewicz, Małgorzata Łazarska, Magdalena Stepczyńska

**Affiliations:** Faculty of Materials Engineering, Kazimierz Wielki University in Bydgoszcz, ul. Chodkiewicza 30, 85-064 Bydgoszcz, Poland; kocisz@ukw.edu.pl (M.K.); kmm@ukw.edu.pl (K.M.); tomakara@ukw.edu.pl (T.K.); lazarska@ukw.edu.pl (M.Ł.); m.stepczynska@ukw.edu.pl (M.S.)

**Keywords:** biopolymer, coffee husks, thermal properties, physico-mechanical properties

## Abstract

This study presents the results of investigations into the properties of a composite made from the commercial biopolymer with varying concentrations of ground coffee husks (CH) at 10, 30, and 50 wt%. Thermal, thermomechanical, physical, and mechanical properties were determined for the composites. Results indicate that the inclusion of CH into the polymer matrix significantly enhances the thermomechanical properties of the obtained composites, particularly in terms of storage modulus at 30 °C. The addition of coffee filler did not alter the characteristic thermal curves. Still, it reduced the thermal resistance of the composites, lowering the degradation temperature by approximately 40 °C compared to the pure polymer. Furthermore, the incorporation of CH introduced an additional stage of mass loss on the thermogravimetric curves, associated with the thermal decomposition of CH. The physico-mechanical properties of the composite depend on the content of the filler. Increasing the coffee content increases the dynamics of water absorption by the composite. It also increases the composite’s stiffness while reducing its tensile and flexural strength. The obtained results suggest that biopolymer-based composites filled with ground CH can be effectively used for manufacturing biodegradable products, with the moisture diffusion behavior indicating susceptibility to degradation that is modulated by the CH content.

## 1. Introduction

Despite efforts to produce environmentally friendly products, more than 98% of all plastics are still derived from fossil fuels, primarily crude oil and natural gas. Global plastic production continues to increase year over year, already exceeding 390 million tons annually. This trend highlights a sustained and growing demand for polymeric materials [1,2,3,4]. At the same time, despite the availability of established recycling methods, the disposal of conventional plastics remains a significant global challenge. It is essential to emphasize that legislative requirements aimed at reducing and ultimately eliminating petroleum-based plastics have driven the polymer processing industry toward the search for sustainable materials derived from eco-friendly raw materials. These alternatives are designed to reduce environmental pollution and simultaneously decrease production costs. Consequently, biodegradable and bio-based polymers have garnered significant interest from both the scientific community and the industrial sector due to their environmentally friendly properties. To meet these objectives, support a sustainable market economy, and address current environmental challenges, biodegradable materials must play an increasingly prominent role in polymer processing and materials engineering [5,6]. In this context, the application of environmentally degradable polymers, such as polylactic acid (PLA), poly(butylene adipate-co-terephthalate) (PBAT), polyhydroxybutyrate (PHB), polybutylene succinate (PBS), and polycaprolactone (PCL), constitutes a promising strategy for reducing the degradation time of polymeric materials while concurrently mitigating environmental pollution. These polymers exhibit the ability to decompose under various environmental conditions, including soil, marine environments, and industrial composting, significantly reducing their environmental footprint [7]. Their biodegradability makes them a viable solution for mitigating waste accumulation and improving waste management efficiency [8]. Despite their presence on the market, biodegradable polymers still face significant barriers that limit their widespread adoption. The primary challenge remains their high production cost, which results from the need to use renewable raw materials and the complexity of the manufacturing processes compared to conventional petrochemical-based polymers [9]. These costs adversely impact their competitiveness relative to conventional materials such as polyethylene (PE) and polypropylene (PP) [5]. One particularly promising strategy to address these challenges, at least partially, is the incorporation of plant-based fillers into polymer matrices. The incorporation of organic fillers, such as plant-derived particulates, not only improves the biodegradability of polymer composites but also contributes to a reduction in the overall production cost [4,10,11,12]. From a materials science standpoint, a particularly promising approach involves the utilization of by-products derived from food processing and agricultural activities as fillers in polymer composites. These solutions align with the principles of the circular bioeconomy by promoting the development of sustainable materials [13,14]. In addition to lignocellulosic waste from the agri-food industry, recent studies highlight the potential of less conventional organic residues, such as keratin derived from discarded human hair, as functional fillers in biodegradable composites. Noyon et al. [15] demonstrated that biocomposites containing hair keratin and graphene oxide exhibit significantly enhanced mechanical strength, thermal resistance, and barrier properties. It has also been observed [16,17] that the use of chemically modified plant-based fillers improves the functional properties of green thermoplastic matrices, further reinforcing the rationale for incorporating diverse waste materials into polymer systems. These approaches align with the principles of the circular economy and sustainable waste management strategies, supporting the development of advanced, environmentally friendly composite materials. Agro-food waste, such as flax fibers, rice husks, jute fibers, almond shells, walnut shells, orange peels, coconut fibers, and sisal fibers, are just a few examples of plant-based fillers extensively studied for incorporation into biopolymer matrices [18,19,20,21,22,23,24,25,26,27]. Green composites reinforced with such plant-based waste materials may exhibit comparable or even superior properties to conventional petroleum-derived polymer composites. Furthermore, the utilization of agro-food waste as polymer fillers can significantly lower overall production costs, reduce or eliminate material toxicity, decrease material density, balance mechanical properties, and, most importantly, minimize environmental impact [28,29]. One of the most abundant agro-food waste materials generated globally is coffee husk. Projections indicate that global coffee production for the 2023/24 season will exceed 170 million bags [30]. Considering that coffee husks (CH) produced as a by-product of coffee processing constitute nearly half the weight of coffee beans, it is evident that vast amounts of waste are generated [4,31]. Although CH is plant-based and generally regarded as environmentally friendly, its application is somewhat limited due to the presence of caffeine, tannins, and other organic compounds. Nevertheless, various efforts have been undertaken to repurpose coffee husks (CH), including their application as fertilizers, animal bedding, fluxing agents in ceramic manufacturing, biomass fuel, and components in water purification systems [32,33,34]. However, given the global scale of coffee production, only a small fraction of available CH is utilized for these purposes. Therefore, in light of current environmental trends promoting resource optimization, the development of effective strategies for utilizing coffee husks as polymer fillers is justified [35]. Recognizing the significant potential of coffee processing waste, including CH [36], recent research has increasingly focused on exploring its applicability as organic fillers in polymer composites. Studies have investigated the properties of polymer composites incorporating CH as a filler in conventional polymers such as polypropylene (PP) [37,38,39], polyethylene (PE), and high-density polyethylene (HDPE) [40,41,42], including those derived from recycled sources [43,44]. These studies emphasize that the incorporation of CH into polymer matrices reduces the consumption of petroleum-based polymers while simultaneously offering environmental benefits. The degree of CH incorporation and its surface modifications significantly influence the resulting composite properties. Researchers have highlighted the advantageous characteristics of CH as fillers, even in cases where a reduction in mechanical strength was observed. Collectively, these studies confirm that the incorporation of CH into polymer composites holds significant potential for applications in the plastics processing industry. Building on these findings, numerous studies have demonstrated that both the amount and morphology of lignocellulosic fillers significantly influence the performance of biodegradable composites. An increase in filler content typically leads to greater stiffness and thermal stability, owing to the rigid structure of plant-based particles and their ability to restrict polymer chain mobility [18,19,20,21]. However, excessive filler content may also cause embrittlement and reduced plasticity, especially when interfacial adhesion is insufficient. Schutc et al. [45] reported that moderate CH loadings of 10–15% improved tensile strength and barrier properties in TPS/CH composites, while higher contents promoted stress concentration and crack initiation. Furthermore, chemical modification of fillers, e.g., by alkaline treatment, can enhance their compatibility with the polymer matrix. This was confirmed by Melyna and Afridana [38], who observed improved interfacial adhesion, increased tensile strength, and reduced water absorption. In contrast, the present study proposes a simplified composite system based on unmodified CH, aiming to minimize technological complexity and preserve full biodegradability. In line with this direction, our earlier work [1] demonstrated that the amount of plant filler affects not only mechanical properties but also the crystallinity and thermal behavior of PLA-based biocomposites. These findings underline the critical role of filler selection and optimization in the design of effective, eco-friendly composites. In recent years, more attention has been directed toward developing composite blends based on biodegradable polymers reinforced with CH. Research has explored the properties of composite materials incorporating CH in biodegradable polymer matrices such as PLA [46,47], thermoplastic starch (TPS) [27,28,29,30,31,32,33,34,35,36,37,38,39,40,41,42,43,44,45,46,47,48,49], and blends of PLA and TPS with CH [45,50] as well as PLA and TPS reinforced with coffee grounds [44] or TPS combined with polycaprolactone (PCL) [4,51]. Other studies have investigated CH-filled composites based on polyhydroxybutyrate (PHB) [52] and polybutylene adipate terephthalate (PBAT) [53]. These studies indicate that the incorporation of CH may have a beneficial effect on the selected mechanical or barrier properties of biodegradable composites, particularly when the filler content and interface compatibility are properly optimized. However, some studies also report reduced toughness or thermal resistance at higher filler loadings, highlighting the importance of formulation balance. In particular, Schutc et al. [45] observed that moderate CH loadings in PLA/TPS blends improved mechanical strength and water vapor barrier properties, while higher filler levels led to increased brittleness. It has also been demonstrated [38] that alkaline treatment of coffee husk (CH) increases its compatibility with polypropylene, resulting in improved interfacial adhesion, higher tensile strength, and reduced water absorption. These findings highlight the importance of filler surface chemistry and content optimization in tailoring the properties of the composite. The results indicate that these eco-friendly composites exhibit well-balanced physico-mechanical properties and significant thermal stability. Moreover, it has been observed that biodegradable polymer composites containing CH can be effectively utilized in the production of compostable packaging and single-use products, including those intended for food contact applications. While the present work builds on previous studies, it introduces a distinct approach by proposing a simplified composite system based on unmodified CH, specifically optimized for cost effectiveness and targeted at short-term packaging applications.

This study aimed to develop and analyze a novel material composition containing CH and a biopolymer, with particular emphasis on its potential application as a packaging composite with increased susceptibility to environmental degradation, manufactured using conventional polymer processing techniques. The proposed solution enables low-cost injection molding production of polymer packaging, including containers with increased wall stiffness.

## 2. Materials and Methods

The materials used in this study comprised coffee husks (CH); a commercial polymer blend MB155—consisting of polylactic acid (PLA) and thermoplastic starch—supplied in granulated form by Azoty S.A., Tarnów, Poland; and a glycerol additive (plant-derived glycerol, 99.5% purity, pharmaceutical grade, viscosity 954 cP) procured from ATE Technology Sp. z o.o., Warsaw, Poland. Previous investigations have demonstrated that the incorporation of an optimal glycerol content in PLA/starch blends significantly enhances the interfacial interactions between these components [47,54,55]. Accordingly, 3 wt% glycerol was incorporated into MB155 to improve the composite’s final properties. The ground coffee husks were sourced from the Research and Development Center for Wood-Based Panels Sp. z o.o. (OBRPPD), Czarna Woda, Poland. To ensure uniformity in particle size, the plant filler was further sieved using a LAB-11-200/UP analytical mixer equipped with 120–35 mesh sieves, yielding a fraction size range of 0.125 mm to 0.5 mm.

### 2.1. Sample Preparation

MB155EF granulates and dried CH with a moisture content below 2% were compounded at polymer-to-filler mass ratios of 90/10, 70/30, and 50/50. The compounding was performed using an EHP-2 × 24M co-rotating twin-screw extruder (Zamak Mercator Sp. z o.o., Skawina, Poland). The barrel temperature profile, from the feed zone to the die, was set as follows: 140, 150, 160, 170, 165, and 160 °C. The screw speed was maintained at a constant 40 rpm. MB155EF granulates were fed through the main hopper, while ground CH was introduced via a synchronized feeder to achieve the desired filler concentration. The extruded strand was cooled in a water bath and subsequently pelletized using a granulator. The resulting granulates from each formulation were redried at 60 °C for 24 h and stored in hermetically sealed bags until the preparation of composite test specimens. Test specimens, including pure polymer and composites, were fabricated via injection molding using an Engel Victory VC120 (ENGEL AUSTRIA GmbH) screw injection molding machine. The temperature profile during processing was set at 140–150–160–170 °C for different barrel zones. Injection pressure time, hold pressure time, and cooling time were set to 3 s, 6 s, and 40 s, respectively. Specimens were manufactured according to EN ISO 527-2 [56]. Following processing, specimens were conditioned under controlled environmental conditions (50% relative humidity and 23 °C) for two weeks prior to testing.

### 2.2. Methods

#### 2.2.1. SEM Analysis of Material Structure

Structural morphology analysis carried out using a Phenom XL scanning electron microscope (SEM) (Thermo Fisher Scientific, Waltham, MA, USA). The surfaces of the analyzed material were obtained through transverse fracturing of the sample. Sample surfaces were prepared by transverse fracturing to expose the internal morphology. Prior to microscopic examination, the fractured surfaces were sputter-coated with a thin layer of gold (Au) to enhance electrical conductivity.

#### 2.2.2. Dynamic Mechanical Analysis (DMA)

Dynamic mechanical analysis (DMA) was carried out using a Q800 dynamic mechanical analyzer (TA Instruments, New Castle, DE, USA). Measurements were performed over a temperature range of 30 to 150 °C at a heating rate of 3 °C/min. Rectangular specimens measuring 60 mm × 10 mm × 4 mm were tested under a strain amplitude of 0.01% and a deformation frequency of 1 Hz.

#### 2.2.3. Differential Scanning Calorimetry (DSC)

Differential scanning calorimetry (DSC) analysis was performed under a nitrogen atmosphere using a Q200 differential scanning calorimeter (TA Instruments, New Castle, DE, USA). Samples weighing approximately 10 mg were tested over a temperature range of −60 to 200 °C at a heating rate of 10 °C/min. The glass transition temperature (Tg), cold crystallization temperature (Tcc), enthalpy of cold crystallization (ΔHcc), melting temperature (Tm), and enthalpy of melting (ΔHm) were determined based on the second heating cycle.

#### 2.2.4. Thermogravimetric Analysis (TGA)

Thermogravimetric analysis (TGA) was performed under a nitrogen atmosphere using a Q500 thermobalance (TA Instruments, New Castle, DE, USA). Composite samples (≈15 mg) and raw CH material (≈9.8 mg) were analyzed from approximately 25 °C to 700 °C at a heating rate of 10 °C/min. The TGA curve was used to determine the T_5%_ value—the temperature at which 5% of the initial sample mass is lost—which was considered an indicator of the thermal stability of the material. Additionally, the residue (R) was recorded. The differential thermogravimetric (DTG) curve, obtained as the first derivative of the TGA curve, was used to identify T_max_ values corresponding to the temperatures at which the maximum degradation rate occurred in each stage of decomposition.

#### 2.2.5. Water Absorption and Thickness Swelling

Water absorption testing was conducted in accordance with ISO 62 [57], by determining the amount of water absorbed after immersion in distilled water at 23 °C. Samples were weighed before immersion and after specified time intervals. Simultaneously, thickness measurements were taken to assess the degree of swelling. Water absorption (WA) and thickness swelling (TS) were calculated using the following Equations (1) and (2):

(1) $$WA=\frac{m_{x}-m_{1}}{m_{1}},$$

where

*m_x_*—mass of the test specimen after immersion for a specified time.

*m*_1_—mass of the test specimen before immersion.

(2) $$TS=\frac{t_{x}-t_{1}}{t_{1}},$$

where

*t*_x_—thickness of the test specimen after immersion for a specified time.

*t*_1_—thickness of the test specimen before immersion.

The experimental WA values were fitted using the short-time approximation of Fick’s second law for one-dimensional diffusion in a slab geometry. The applied equation was (3):

(3) $$M_{t}=M_{\infty }\left(\frac{4}{L}\sqrt{\frac{Dt}{\pi }}\right)$$

where

*M*_t_—water uptake at time t.

*M*_∞_—saturation level.

*L*—thickness of the sample.

*D*—effective diffusion coefficient.

Fitting was performed using non-linear regression.

#### 2.2.6. Mechanical Testing

Tensile—including tensile modulus, tensile strength, and elongation at break—as well as bending properties—comprising bending modulus, bending strength, and maximum deflection at break—were evaluated using testing machine, type Instron 3367 (Instron, Norwood, MA, USA) in accordance with ISO 527-1 [58] and ISO 178 [59]. Ten replicates were tested for each measurement. All tests were conducted at room temperature (20 °C) and a controlled relative humidity of 50%.

#### 2.2.7. Statistical Analysis

The obtained data were statistically analyzed using Statistica version 13. A one-way analysis of variance (ANOVA) was conducted to determine the significance of the effect of filler content on the mechanical properties of the composites. Tukey’s post hoc test was also applied to assess the statistical significance of differences between the mean values of the composite properties.

## 3. Results and Discussion

### 3.1. SEM Analysis of Material Structure

**Figure 1:**
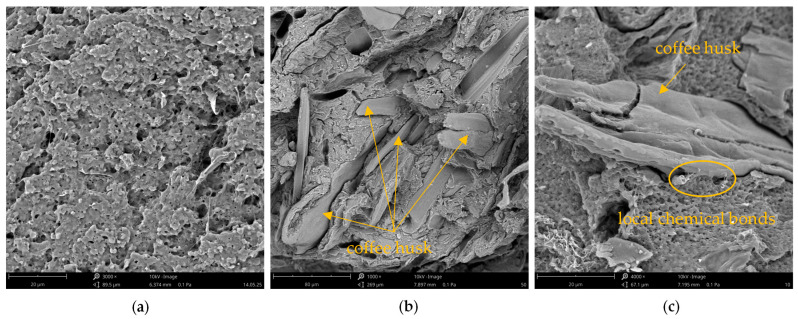
SEM images of sample cross-sections: (**a**) polymer without filler (magnification × 3000), (**b**) composite with 50% CH (magnification × 1000), and (**c**) interface between polymer and filler phases (magnification × 4000).

Scanning electron microscopy (SEM) images in Figure 1 show the cross-sectional morphology of the fractured polymer and coffee husk (CH)-filled composite.

The cross-sectional image of the neat polymer sample (Figure 1a) reveals a relatively uniform, fine-grained morphology characteristic of plasticized starch matrices. The absence of visible inclusions or major irregularities indicates a proper processing method. However, the observed microporosity within the structure may be attributed to the migration of water or plasticizer during the cooling process, a phenomenon well-documented in starch-based systems [60]. In the case of the sample shown in Figure 1b, containing 50 wt% CH filler, numerous filler particles with distinctly irregular shapes are distributed throughout the matrix volume. The structure of the filler is complex. The particles exhibit sharp, brittle surfaces and vary in size, which is a result of the mechanical grinding of the husks before their incorporation into the composite. The filler appears to be randomly arranged, with no preferential orientation, which is typical for composites formed by injection molding or hot pressing [61]. The degree of mixing suggests a relatively uniform dispersion of particles; however, local agglomerates or areas of increased filler concentration can be observed, which may lead to non-uniform stress distribution within the material [62]. A significant aspect visible in Figure 1b,c is the quality of the bonding between the polymer phase and the filler. In many areas, clear gaps can be seen around the CH particles, which suggests insufficient wetting of the filler surface by the polymer. This may indicate that the dominant type of interfacial adhesion is physical bonding (mechanical interlocking), and in some cases, so-called keying effects may occur, resulting from the mechanical anchoring of the polymer within the micropores of the filler surface [40]. Figure 1c provides a detailed view of the polymer–filler interface in the transition zone. In select regions, the polymer matrix intimately surrounds the husk particles without detectable voids, implying the presence of localized chemical bonding. These bonds likely arise from interactions between hydroxyl groups present in both the polymer and the lignocellulosic constituents of CH (cellulose, hemicellulose, and lignin). Although such chemical interactions are limited and spatially inconsistent, they may locally enhance interfacial adhesion and contribute to improved mechanical performance of the composite. Overall, SEM analysis demonstrates that filler particles exhibit varying degrees of embedding within the polymer matrix. While some particles are well-integrated, the frequent presence of interfacial voids may detrimentally impact the mechanical properties of the composites, particularly with regard to stress transfer efficiency at the filler–matrix interface [63].

### 3.2. Thermomechanical Properties

**Figure 2:**
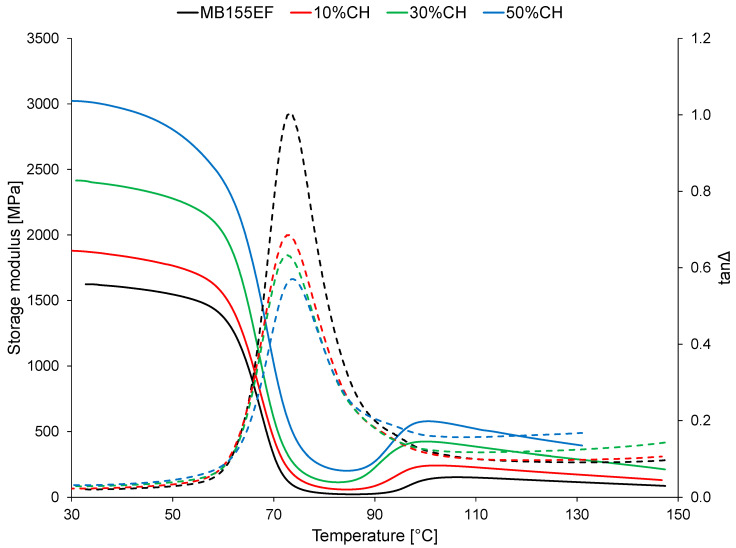
Thermomechanical (DMA) curves of the tested materials; solid line—storage modulus; dashed line—tanΔ.

The thermomechanical curves of the tested materials are presented in Figure 2. The values of the storage modulus determined at selected temperatures are summarized in Table 1.

The thermomechanical behavior of the MB155EF material, as illustrated in Figure 2, is characteristic of thermoplastic polymers to which this material belongs. The storage modulus of the neat MB155EF sample at 30 °C (E′_30_) was 1624 MPa. This value remained relatively stable up to approximately 50 °C, where the measured modulus (E′_50_) was 1546 MPa. Beyond this temperature, a pronounced decrease in storage modulus occurred, corresponding to the glass-to-rubber transition typical of thermoplastic materials. The modulus decreased significantly, reaching 26 MPa at 90 °C (E′_90_).

Within the temperature range of 50–90 °C, a thermomechanical glass transition (T_gDMA_) was observed, identified as the peak in the tanΔ curve. For MB155EF, the T_gDMA_ was determined to be 73.2 °C. Furthermore, the DMA curve of the MB155EF sample exhibited a characteristic secondary increase in storage modulus in the range of 90–140 °C, attributed to cold crystallization. The formation of a crystalline phase in this temperature range, possessing inherently different mechanical properties, contributes to the slight increase in modulus. In the case of MB155EF, E′ at 120 °C (E′_120_) increased to 133 MPa.

The incorporation of the filler into the polymer matrix did not substantially modify the shape of the thermomechanical curves of the resulting composites. Moreover, the addition of the filler had no significant impact on the glass transition behavior, as the T_gDMA_ values for all tested materials were comparable, with differences not exceeding 1.0 °C. These findings suggest that coffee husk filler does not alter the mobility of the polymer chains; consequently, the observed changes in the DMA results are primarily attributable to the physical properties of the filler, which increase the stiffness of the composites.

The presence of the filler exerted a pronounced effect on the absolute values of the storage modulus. Most notably, a distinct increase in E′_30_ was recorded with the addition of filler particles. The increase in stiffness was directly proportional to the filler content. The addition of 10 wt% CH filler raised E′_30_ to 1879 MPa, approximately 15% higher than that of the MB155EF matrix. A 50 wt% filler loading increased E′_30_ to 3023 MPa, nearly double the value observed for the neat polymer. This indicates that the composite samples exhibited significantly higher stiffness.

The increase in stiffness was also reflected in a substantial decrease in tanΔ, the damping factor. The tanΔ peak dropped from 1.005 for MB155EF to 0.5700 for the sample containing 50 wt% filler (50%CH). This suggests a lower energy dissipation capability in the composites, indicating that components manufactured from these materials will exhibit reduced vibration-damping performance. Since the studies showed no significant effect of the filler on the mobility of polymer chains, it can be assumed that the observed decrease in tanΔ is caused by other phenomena. Fillers increase the stiffness of the composite, which reduces the material’s ability to undergo viscoelastic deformations, which are responsible for damping vibrations. Reduced deformation corresponds to lower energy dissipation as heat, thereby lowering the damping capability. Furthermore, insufficient interfacial bonding between the filler and polymer matrix may induce micro-cracking or interphase slippage, which fails to effectively dissipate energy. The resulting poor stress transfer can further reduce damping. Finally, filler agglomerates may act as localized stiffeners or structural defects, generating heterogeneities that undermine the composite’s intrinsic damping mechanisms.

A reduced tanΔ value indicates less capability to absorb and dissipate vibrational energy, which might be unfavorable for applications requiring vibration or impact damping (e.g., automotive interiors). Lower damping may still be acceptable, especially if acoustic performance is sufficient and lightweighting via biofillers is prioritized. Enhanced stiffness and structural stability may be advantageous in static or semi-dynamic applications where higher stiffness and shape retention under load are required. Higher stiffness and better dimensional stability associated with higher E’ and lower tanΔ can be beneficial in applications where mechanical precision or load-bearing capacity is prioritized over damping, such as structural panels, packaging components, and furniture parts.

A further increase in storage modulus was observed in the temperature region associated with cold crystallization. The rising E′_120_ values with increasing filler content can be attributed primarily to the greater proportion of rigid material (coffee husk) in the composite. This interpretation is supported by DSC results presented later in this article, which did not indicate a significant enhancement of cold crystallization intensity due to the presence of the filler. On the other hand, the addition of the filler did not affect the glass transition behavior of the composites. The T_gDMA_ values obtained for all tested materials were comparable, with differences not exceeding 1.0 °C.

### 3.3. Thermal Properties

**Figure 3:**
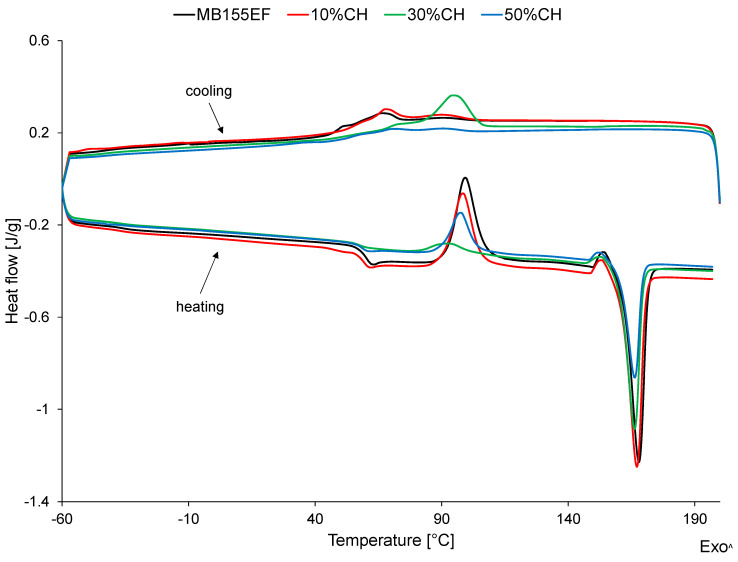
Thermal curves of the tested materials.

The thermal curves of the tested materials are presented in Figure 3, with the results from the second heating cycle summarized in Table 2. The cooling curves of the examined samples reveal transitions related to crystallization and the glass transition processes. However, due to the thermal overlap of these effects, it was not possible to determine their exact transition temperatures with sufficient clarity. For this reason, those values are not included in Table 2. Nevertheless, the presented cooling curves will be referenced later in this article to support and explain specific aspects of the thermal analysis and interpretation of results.

The thermal curve recorded during the second heating of the MB155EF sample exhibited thermal events corresponding to the glass transition, cold crystallization, reorganization of the crystalline phase, and melting of the crystalline phase.

The glass transition temperature (T_g_) of the MB155EF sample was determined to be 60.1 °C. This value differs significantly from the glass transition temperature determined by dynamic mechanical analysis (T_gDMA_), which is expected. Discrepancies between T_g_ values obtained by DSC and DMA are well-documented in the literature. Typically, T_g_ values from DSC are lower than those from DMA due to the fundamental differences in measurement principles. In DSC, T_g_ is determined from the change in specific heat associated with a density shift during the transition from the glassy to the rubbery state. In contrast, DMA detects T_g_ based on mechanical property changes occurring during the same transition.

The cold crystallization temperature (T_cc_) for MB155EF was recorded at 99.3 °C. This phenomenon is typical for certain polymers and results from increased chain mobility above Tg. The thermal energy supplied during heating allows polymer chains to rearrange toward a more thermodynamically stable, crystalline structure. Before the melting peak, an additional exothermic signal was observed, likely associated with crystalline phase reorganization—specifically, a transition from the α′ to the more stable α polymorphic form.

The melting temperature (T_m_) was determined to be 167.2 °C, corresponding to the melting of crystalline structures formed both during the cooling phase and through cold crystallization. Due to the lack of reference enthalpy values for 100% crystalline MB155EF, it was not possible to calculate the degree of crystallinity. However, analysis of the measured enthalpy of cold crystallization (ΔH_cc_) and melting (ΔH_m_), combined with the cooling curve profile, indicates that the tested material contained crystalline domains and can therefore be classified as semicrystalline.

The incorporation of CH filler into the MB155EF matrix did not alter the general shape of the thermal curves. All thermal events observed in the neat polymer matrix were retained in the composites, with no additional thermal transitions attributable to the presence of the filler detected.

A slight decrease in T_g_ was observed with increasing filler content; however, the reduction did not exceed 2.0 °C and is thus negligible from an application standpoint. No clear linear relationship was observed between filler content and cold crystallization temperature (T_cc_) or its intensity (ΔH_cc_).

For 10%CH and 50%CH, a minor decrease in both T_cc_ and ΔH_cc_ was noted in filler-containing materials. This reduction in crystallization behavior, evident in both the cooling and second heating curves, was accompanied by a diminished intensity of melting endotherms, which may suggest a decrease in the crystalline phase content. However, it is more plausible that the attenuated thermal signals primarily result from the reduced polymer phase fraction in the composites due to the increasing volume fraction of filler.

A different effect of the filler was observed for 30% CH. In the case of this material, a clear effect of cash scales on the course of the material crystallization process was observed and more precisely on the course of crystallization of the PLA phase of the tested composite. A clear exothermic peak associated with the formation of the crystalline phase was obtained on the cooling curve, which was accompanied by a large reduction in the cold crystallization peak on the heating curve. Notably, no significant changes were detected in the melting temperature of the crystalline phase for the 30% CH composite. These observations indicate that, unlike the other tested materials, the crystalline phase in this composite is predominantly formed during the cooling process, suggesting that the presence of the filler exerts a measurable influence on the crystallization kinetics.

**Figure 4:**
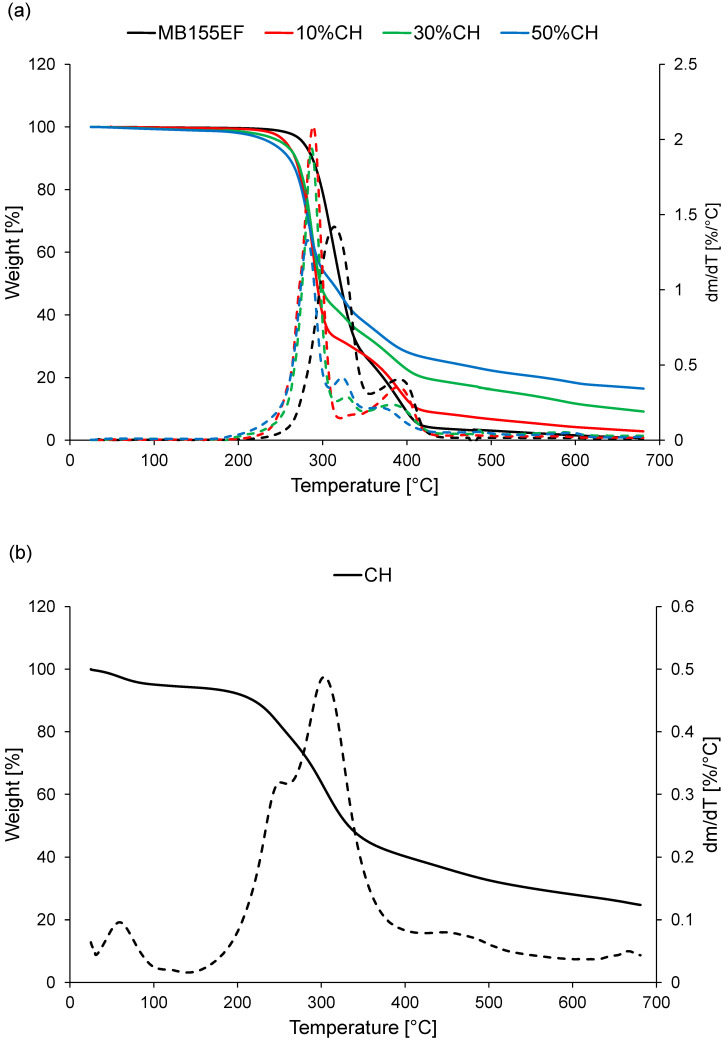
Thermogravimetric curves: (**a**) tested polymer-based materials; (**b**) CH. Solid line—mass; dashed line—derivative (dm/dT).

Figure 4a presents the thermogravimetric (TGA) curves of the tested composite materials, while Figure 4b shows the thermal decomposition behavior of the CH filler. The corresponding TGA results are summarized in Table 3.

Firstly, an increase in filler content led to a decrease in the thermal degradation temperature of the composites. The addition of 10 wt% filler reduced the degradation temperature (T_d_) by approximately 20 °C. When the filler content was increased to 50 wt%, the T_d_ was lowered by around 40 °C compared to the neat MB155EF sample. This reduction in thermal stability is attributed to the significantly lower thermal resistance of the filler. Plant-based organic fillers typically exhibit T_d_ values below 220 °C, which are considerably lower than those of most polymer matrices. This was also the case for the applied CH filler, for which thermogravimetric analysis indicated a T_d_ of approximately 195 °C (Figure 4b). As a result, the higher the filler content in the composite, the earlier the 5% mass loss (taken as an indicator of thermal stability) occurred. Nonetheless, the reduction in thermal stability to approximately 240 °C is not of practical concern, as this temperature still significantly exceeds typical service temperatures for polymer composites.

Secondly, the incorporation of the filler introduced an additional mass loss stage on the thermogravimetric curve, corresponding to the thermal degradation of the CH. This stage occurred in the range of 300–350 °C and became more prominent with increasing filler content. The origin of this degradation step was confirmed by the TG curve of the filler alone (Figure 4b), which showed its main degradation phase occurring within the same temperature range as that observed in the composites.

Thirdly, the addition of the filler resulted in a significant residue remaining after thermal decomposition. The amount of residue correlated with the filler content in the composite, ranging from 2.9% for the 10%CH sample to 16.5% for the 50%CH sample. This residue results from the decomposition characteristics of the CH, which does not fully decompose up to 700 °C. The TG analysis of the filler revealed a final residue of 25.7% after testing, confirming its contribution to the residual mass observed in the composites.

### 3.4. WA and TS

**Figure 5:**
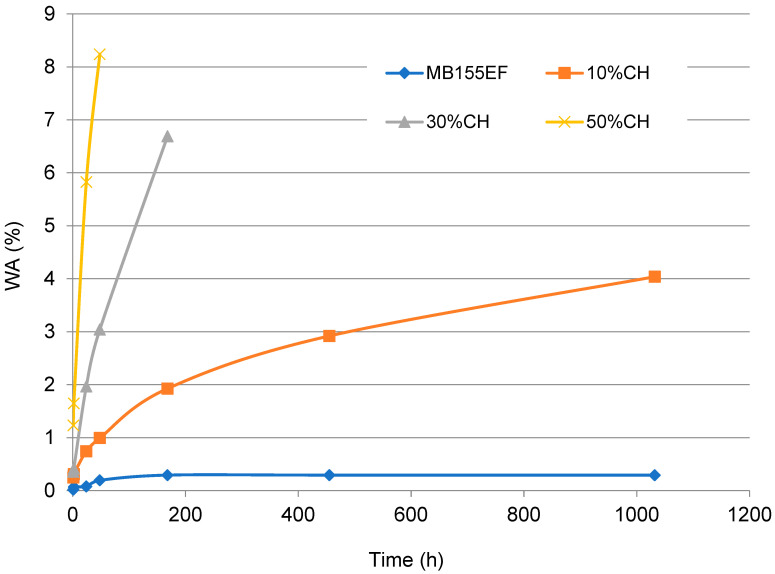
Effect of filler content on the WA of tested materials.

**Figure 6:**
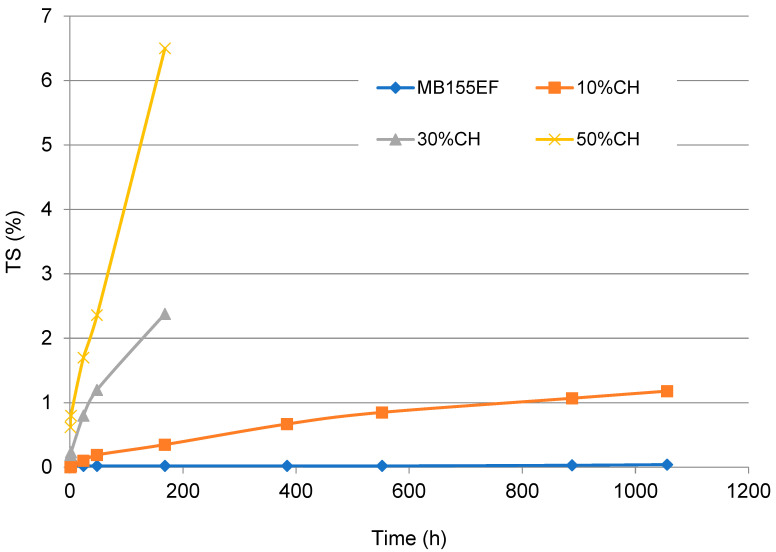
Effect of filler content on the TS of tested materials.

In this study, the effects of water immersion time on WA (Figure 5) and TS (Figure 6) were investigated for composites based on the MB155EF polymer filled with ground CH as well as for the neat polymer. The results demonstrated a significant dependence of both parameters on immersion time and filler content.

The pure polymer exhibited minimal water absorption, attributable to its intrinsically hydrophobic nature. The incorporation of CH filler, which is rich in hydroxyl groups capable of forming hydrogen bonds with water molecules, resulted in a substantial increase in water uptake. For example, the composite containing 10 wt% filler demonstrated approximately a ninefold increase in WA after 24 h of immersion compared to the neat polymer. Increasing the filler content to 30 wt% and 50 wt% resulted in WA values approximately 3-fold and 8-fold higher, respectively, relative to the 10%CH composite. The pronounced increase in WA observed for the 50%CH sample underscores the dominant influence of the hydrophilic nature of CH, which facilitates accelerated water diffusion and earlier saturation of the composite material.

A similar trend was observed in the TS behavior. The neat polymer exhibited minimal dimensional changes, indicating high stability under aqueous conditions. In contrast, the addition of filler led to progressively greater swelling. The composite containing 10 wt% CH showed a fivefold increase in TS after 24 h of immersion compared to the neat polymer. Increasing the filler content to 30 wt% and 50 wt% resulted in approximately 8-fold and 17-fold increases in TS, respectively. This increase in TS is directly correlated with the higher water uptake observed in the composites. Additionally, it is influenced by diminished interfacial adhesion between the polymer matrix and the filler. The weaker interfacial bonding facilitates the formation of microcracks, thereby promoting enhanced water ingress and swelling within the composite structure.

To further characterize the water transport behavior in the composites, the experimental WA data were fitted using a simplified Fickian diffusion model applicable for short time intervals. Assuming a sample geometry (thickness 4 mm) and one-dimensional moisture diffusion, the effective diffusion coefficients (D) were estimated for composites containing 10 wt%, 30 wt%, and 50 wt% CH. The results revealed a gradual increase in the diffusion coefficient from 2.69 × 10^−11^ m^2^/s for 10 wt% CH to 3.03 × 10^−11^ m^2^/s for 50 wt% CH. This trend is consistent with the observed increase in WA and TS, suggesting that higher filler content enhances water transport, possibly due to increased matrix porosity or filler–matrix interfacial regions. Although this model is limited to early-stage absorption and assumes constant diffusivity, it provides a useful approximation of moisture uptake behavior and confirms the role of filler content in governing environmental interactions.

### 3.5. Mechanical Properties

**Figure 7:**
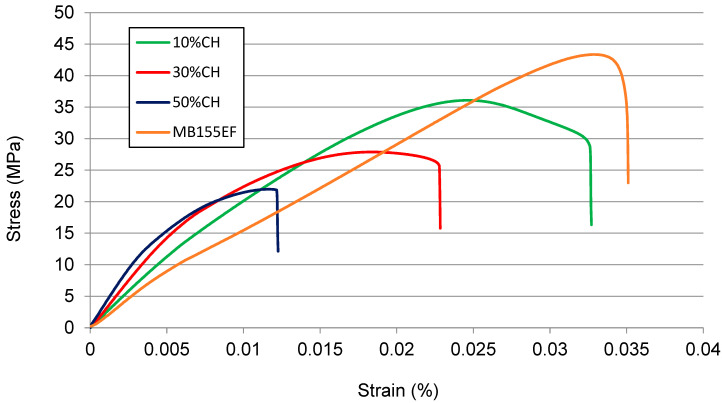
The effect of filler content on the stress–strain relationship under tensile load of the tested composites.

**Figure 8:**
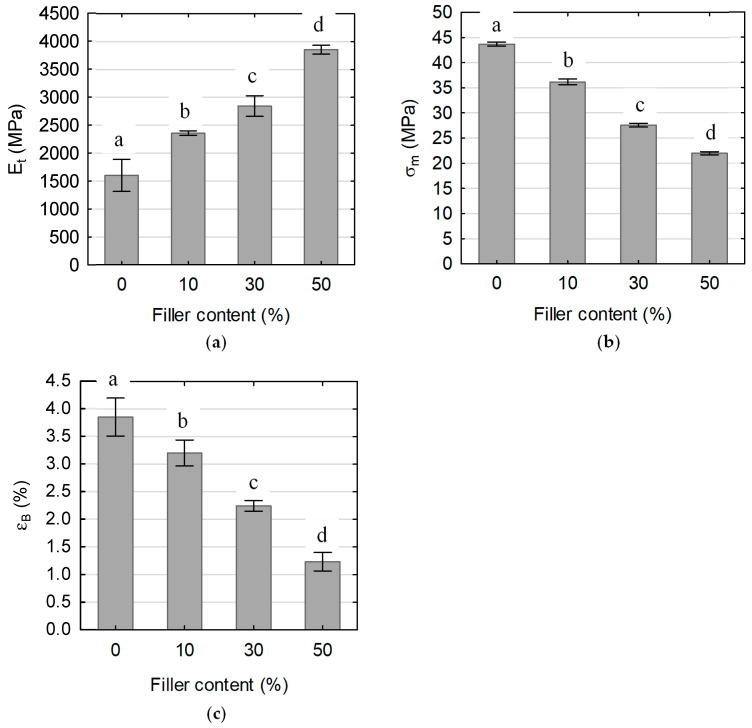
Effect of filler content on tensile properties of tested composites: (**a**) tensile modulus, (**b**) tensile strength, and (**c**) elongation at break. Mean values with the same letter for the given property are not statistically different at the 5% significance level.

**Figure 9:**
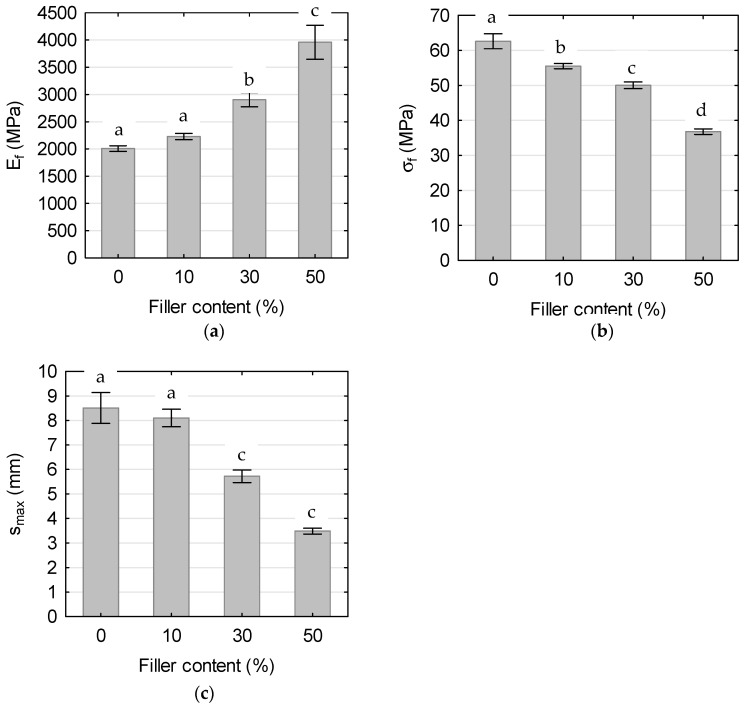
Effect of filler content on bending properties of tested composites: (**a**) bending modulus, (**b**) bending strength, and (**c**) maximum deflection. Mean values with the same letter for the given property are not statistically different at the 5% significance level.

Figure 7, Figure 8 and Figure 9 show the stress–strain relationships (Figure 7) and mean values of the tensile modulus (Figure 8a), tensile strength (Figure 8b) and elongation at break (Figure 8c), the flexural modulus (Figure 9a), flexural strength (Figure 9b), and maximum deflection (Figure 9c) of tested WPCs with CH. Error bars represent one standard deviation based on ten specimens. One-way analysis of variance (ANOVA) was conducted to determine the significance of the effects of filler content on the WPC mechanical properties. Results of this analysis show that all mechanical properties vary significantly with filler content.

#### 3.5.1. Tensile Properties

The tested composites were subjected to uniaxial tensile loading, and the resulting stress–strain curves are presented in Figure 7. The neat MB155EF polymer exhibited an approximately linear stress–strain response, achieving a maximum tensile strength of approximately 43 MPa. The addition of 10 wt% CH filler induced a slight nonlinear behavior in the stress–strain curve and led to a reduction in maximum tensile strength to approximately 36 MPa. Further increases in filler content to 30 wt% and 50 wt% resulted in a more pronounced nonlinear response and progressive weakening of the composite, with maximum tensile strengths decreasing to 28 MPa and 22 MPa, respectively.

Figure 8 presents a comparison of selected tensile properties for the neat MB155EF polymer and composites with varying CH filler content. The incorporation of ground CH significantly enhanced the elastic modulus of the material (Figure 8a). Specifically, the composite containing 10 wt% filler exhibited a modulus of 1602 MPa, representing a 47% increase relative to the neat polymer matrix. Further increments in filler content to 30 wt% and 50 wt% resulted in additional stiffness improvements, with modulus increases of approximately 21% and 63%, respectively. This enhancement is attributed to the reinforcing effect of the rigid CH particles, which improve the composite’s resistance to deformation. As noted earlier, the incorporation of CH filler led to a reduction in tensile strength (Figure 8b). The composite with 10 wt% filler showed a 17% decrease in strength compared to the neat polymer, reaching a maximum stress of 36 MPa. Increasing the filler content to 30 wt% and 50 wt% led to further proportional declines in tensile strength, with reductions of 24% and 39%, respectively. This deterioration is primarily attributed to inadequate interfacial adhesion between the polymer matrix and filler particles as well as the formation of structural defects such as voids and microcracks, which act as stress concentrators and promote premature failure A similar trend was observed in elongation at break (Figure 8c). At 10 wt% CH, elongation at break decreased to 3.2%, reflecting a 17% reduction compared to the neat polymer. Increasing the filler content to 30 wt% and 50 wt% further reduced elongation by approximately 30% and 61%, respectively. This reduction indicates increased brittleness of the composites, where a higher concentration of rigid CH particles facilitates microcrack initiation and propagation, thereby diminishing the material’s capacity to sustain tensile deformation.

#### 3.5.2. Bending Properties

The results of flexural property testing for the analyzed materials are presented in Figure 9. The observed trends as a function of filler content closely parallel those obtained from tensile testing. Both the presence and increasing concentration of CH filler contributed to a rise in flexural modulus (Figure 9a), accompanied by decreases in flexural strength (Figure 9b) and maximum deflection at break (Figure 9c). The composite containing 10 wt% filler exhibited a flexural modulus that was 11% higher than that of the neat polymer, reaching 2228 MPa. Increasing the filler content to 30 wt% led to a 30% increase in modulus, while the 50 wt% filler composite showed a 78% increase, reaching nearly 4000 MPa. Notably, for the composites, the flexural modulus values were very similar to those determined in tensile tests, with relative differences not exceeding 6%. In contrast, the difference for the neat polymer was more pronounced, around 25%.

Flexural strength was also strongly influenced by filler content. The addition of 10 wt% CH reduced flexural strength to 55 MPa, which corresponds to an 11% decrease compared to the neat polymer. Further increases in filler content to 30 wt% and 50 wt% resulted in reductions of 10% and 34%, respectively. As expected, flexural strength values were, on average, approximately 60% higher than those obtained in the tensile tests.

The incorporation of 10 wt% filler resulted in a slight reduction in maximum deflection at break, decreasing by approximately 5%. However, this effect became more pronounced at elevated filler loadings, with the 30 wt% and 50 wt% composites exhibiting reductions of 29% and 57%, respectively. These findings reflect the progressive embrittlement of the material associated with increasing concentrations of rigid filler particles.

## 4. Conclusions

The incorporation of coffee husk (CH) filler into the MB155EF polymer matrix markedly enhanced the thermomechanical properties of the resulting composites, most notably the storage modulus at 30 °C. Specifically, the composite with 50 wt% CH exhibited a storage modulus nearly double that of the neat MB155EF polymer.

The addition of CH to MB155EF did not alter the overall shape of the thermal curves. All thermal events observed in the neat matrix were still present in the composites, and no additional thermal transitions associated with the filler were detected. A slight decrease in glass transition temperature was observed with increasing filler content; however, this decrease did not exceed 2.0 °C. A minor reduction in the cold crystallization temperature was also noted for the filled materials, while the melting temperature remained unaffected by the presence of the filler.

The thermal stability of the composites decreased with the incorporation of CH filler. At the highest filler loading of 50 wt%, the degradation temperature (T_d_) of the composite was reduced by approximately 40 °C relative to the neat MB155EF matrix. Furthermore, an additional mass loss stage appeared in the thermogravimetric analysis (TGA) curves of the composites, attributable to the thermal degradation of the CH filler. Despite this reduction, the thermal stability of around 240 °C remains sufficiently high for most typical service conditions of polymer composites, thus limiting practical concerns.

The neat MB155EF polymer exhibited very low water absorption and negligible swelling, coupled with low stiffness and moderate mechanical strength. Incorporation of CH filler markedly increased both water uptake and swelling of the composites, with these effects becoming more pronounced as the filler content rose. Importantly, the filler concentration also influenced the kinetics and extent of physical degradation upon water immersion; composites with higher filler loadings exhibited earlier onset of visible deterioration, including cracking and fragmentation. This behavior suggests that materials with elevated CH content can achieve a balance between maintaining satisfactory functional performance and promoting controlled physical degradation in aqueous environments, a desirable attribute for packaging applications targeting reduced environmental persistence.

The incorporation of CH particles into the polymer matrix led to increased stiffness of the composites, accompanied by a concomitant decrease in mechanical strength. As the filler content increased, a significant enhancement in the elastic modulus was observed under both tensile and flexural loading, indicating improved rigidity of the resultant packaging material. Although reductions in tensile and flexural strength were noted, these decreases are deemed acceptable within the context of packaging applications, where enhanced stiffness and biodegradability typically take precedence over maximal mechanical strength.

## Figures and Tables

**Table 1:** Results of thermomechanical analysis.

Sample	E′_30_ [MPa]	E′_50_ [MPa]	E′_90_ [MPa]	E′_120_ [MPa]	tanΔ	T_gDMA_ [°C]
MB155EF	1624	1546	27	133	1.005	73.2
10%CH	1879	1765	86	200	0.6849	73.1
30%CH	2415	2278	226	335	0.6318	72.8
50%CH	3023	2805	273	457	0.5700	73.7

**Table 2:** Results of differential scanning calorimetry (DSC) analysis.

Sample	T_g_ [°C]	T_cc_ [°C]	ΔH_cc_ [J/g]	T_α’→α_ [°C]	ΔH _α’→α_ [J/g]	T_m_ [°C]	ΔH_m_ [J/g]
MB155EF	60.1	99.3	24.0	154.0	1.3	167.2	32.6
10%CH	59.3	98.4	19.9	153,2	1.5	167.1	31.5
30%CH	58.2	93.2	4.5	153.0	1.3	166.2	25.2
50%CH	58.5	97.6	11.2	152.6	1.1	166.3	17.3

**Table 3:** Results of thermogravimetric analysis (TGA).

Sample	T_d_ [°C]	T_max1_ [°C]	T_max2_ [°C]	T_max3_ [°C]	R [%]
MB155EF	277.0	313.4	-	395.9	0.7
10%CH	256.5	288.6	333.8	390.8	2.9
30%CH	251.8	287.0	329.8	387.7	9.2
50%CH	238.4	282.1	323.0	367.5	16.5
CH	195.4	250.5	303.2	469.8	24.8

## Data Availability

The original contributions presented in this study are included in the article. Further inquiries can be directed to the corresponding author.
